# Swin Transformer Based Recognition for Hydraulic Fracturing Microseismic Signals from Coal Seam Roof with Ultra Large Mining Height

**DOI:** 10.3390/s25216750

**Published:** 2025-11-04

**Authors:** Peng Wang, Yanjun Feng, Xiaodong Sun, Xing Cheng

**Affiliations:** 1 CCTEG Coal Mining Research Institute, Beijing 100013, China; cristiarno@163.com (Y.F.); cctegsunxiaodong@163.com (X.S.); 2 School of Information and Communication Engineering, Beijing Information Science and Technology University, Beijing 100192, China; cheng@bistu.edu.cn

**Keywords:** coal mine microseismic, hydraulic fracturing, microseismic sensors, frequency slice wavelet transform, deep learning

## Abstract

Accurate differentiation between microseismic signals induced by hydraulic fracturing and those from roof fracturing is vital for optimizing fracturing efficiency, assessing roof stability, and mitigating mining-induced hazards in coal mining operations. We propose an automatic identification method for microseismic signals generated by hydraulic fracturing in coal seam roofs. This method first transforms the microseismic signals induced by hydraulic fracturing and roof fracturing into time-frequency feature images using the Frequency Slice Wavelet Transform (FSWT) technique, and then employs a sliding window (Swin) Transformer network to automatically identify and classify these two types of time-frequency feature maps. A comparative analysis is conducted on the performance of three methods—including the signal energy distribution method, Residual Network (ResNet) model, and VGG Network (VGGNet) model—in identifying microseismic signals from hydraulic fracturing in coal seam roofs. The results demonstrate that the Swin Transformer recognition model combined with FSWT achieves an accuracy of 92.49% and an F1-score of 92.96% on the test set of field-acquired microseismic signals from hydraulic fracturing and roof fracturing. These performance metrics are significantly superior to those of the signal energy distribution method (accuracy: 64.70%, F1-score: 64.70%), ResNet model (accuracy: 88.04%, F1-score: 89.24%), and VGGNet model (accuracy: 88.47%, F1-score: 89.52%). This advancement provides a reliable technical approach for monitoring hydraulic fracturing effects and ensuring roof safety in coal mines.

## 1. Introduction

To ensure the safe and efficient exploitation of coal resources, regional hydraulic fracturing technology is employed to weaken extremely thick and hard rock strata, thereby reducing the intensity of rock pressure manifestations [1,2,3,4]. During the process of hydraulic fracturing in coal seam roofs, the efficient and automatic identification of diverse microseismic signals serves as the fundamental basis and critical prerequisite for accurate hydraulic fracture network localization, source mechanism analysis, and passive seismic velocity imaging [5]. However, microseismic signals induced by numerous hydraulic fracturing events during monitoring are easily masked by signals from other microseismic sources, such as roof fracturing, shearer coal cutting, directional drilling, and vibrations from ground trucks. These interfering signals exhibit similar time-domain waveforms and partially overlapping frequency spectra with those generated by hydraulic fracturing. Therefore, accurate, efficient, and automatic identification of hydraulic fracturing-related microseismic signals in coal mines is of great significance for fracture network localization and fracturing effectiveness evaluation [6,7].

Scholars have conducted extensive research on the identification of natural microseismic waveforms and proposed various methodologies, among which the Akaike Information Criterion (AIC) [8,9] and the Short-Term Average/Long-Term Average (STA/LTA) algorithm [10] are the most widely adopted. While these methods demonstrate satisfactory identification performance for high signal-to-noise ratio (SNR) microseismic signals, their efficacy degrades significantly when processing low signal-to-noise ratio signals. To address this limitation, researchers have explored strategies involving the combination of multiple techniques or the refinement of existing methods [5,11] to achieve automated microseismic waveform identification. Li et al. [5] proposed a STA/LTA algorithm based adaptive sliding window update method, which leverages the similarity characteristics of acoustic emission waveforms generated during coal-rock fracture. Within a dynamically adjusted adaptive sliding window framework, they established a waveform cross-correlation function model, thus realizing the automated identification of a large number of acoustic emission events during coal-rock failure processes. Zhu et al. [11] developed a first-arrival picking method that integrates optimized wavelet packet threshold denoising with an improved AIC algorithm, specifically designed for the frequency-domain characteristics of hydraulic fracturing-induced microseismic signals. This approach substantially enhanced the conventional AIC algorithm by proposing an Improved AIC (IAIC) algorithm that incorporates both rapid localization and precise extraction steps, thereby achieving accurate P-wave first-arrival time picking for microseismic signals during coal seam hydraulic fracturing. These identification strategies predominantly rely on one or multiple statistical features; however, the determination of feature thresholds often requires manual selection. This inherent limitation poses significant challenges in identifying an optimal threshold that maximizes both recall and precision rates simultaneously.

In the field of time-frequency analysis for mine microseismic signal identification, common methodologies include the Short-time Fourier Transform (STFT), Wavelet Transform (WT), and its extended form Wavelet Packet Transform (WPT), among others [12]. However, these approaches face certain limitations, such as the difficulty in the optimal selection of wavelet bases and appropriate decomposition levels; improper selection may lead to deviations or even inaccuracies in the analysis results. To address this constraint, Zhao et al. [13] introduced an advanced time-frequency analysis method, namely the Frequency Slice Wavelet Transform (FSWT), into the analysis of microseismic signals. By integrating frequency slice functions, FSWT not only enables simultaneous feature extraction in both time and frequency domains, realizing the precise characterization of local signal attributes, but also supports signal reconstruction within arbitrary frequency bands through inverse transformation. Moreover, its time-frequency resolution can be flexibly adjusted by modifying parameters according to specific requirements. Through comparative studies of typical blast vibration signals and rock mass microseismic signals, this method revealed significant differences in their time-frequency characteristics and energy distribution patterns, providing valuable guidance for accurate microseismic signal identification.

Beyond time-frequency analysis tools, traditional signal identification methods such as energy spectrum analysis—which depend on handcrafted features (e.g., peak energy density, frequency band occupancy) for signal classification [13]—exhibit one critical limitations in the context of coal seam roof hydraulic fracturing applications: they are incapable of capturing non-linear, fine-grained time-frequency correlations, particularly between highly similar signals (e.g., hydraulic fracturing versus roof fracturing signals), resulting in misclassification when energy distributions overlap.

Since its initial proposal in 2015 [14], deep learning technology has catalyzed a paradigm shift from informatization to intelligentization in multiple fields, achieving breakthroughs with performance that significantly outperforms traditional methods, particularly in computer vision, natural language processing, and speech recognition. Wang et al. [15] argued that intelligentization represents a pivotal future research direction for the mining industry. In recent years, deep learning techniques have been introduced into the field of seismology, yielding a series of prominent application achievements [16,17,18,19,20,21,22,23,24,25,26]. The Convolutional Neural Network (CNN), a widely adopted architecture in deep neural networks, excels in image recognition tasks due to its inherent convolutional properties. Currently, CNNs are extensively employed in research areas such as microseismic waveform identification [19,20,21,22,23], seismic phase arrival picking [24,25], and microseismic time-series prediction [26], exhibiting performance superior to that of conventional methods.

The Residual Neural Network (ResNet) effectively addresses the issues of gradient vanishing and explosion during training in ultra-deep networks by incorporating skip connections into its architecture, thereby significantly enhancing the performance of deep models [27]. Building on this advancement, Yang et al. [21] proposed an automatic recognition and classification model for mine microseismic signals based on ResNet18, which enables accurate distinction between microseismic signals and blast signals. Zhao et al. [22] developed a mine microseismic signal recognition model based on the VGG network proposed by the Visual Geometry Group (VGG) of the University of Oxford [28]. This network, constructed with a series of consecutive small convolutional kernels and max-pooling layers, achieves high accuracy in recognizing microseismic signals from nine types of events, including rock fracturing, blasting activities, and background noise during mining processes. However, mainstream CNN-based models such as ResNet and VGGNet possess inherent limitations that impede their application to the identification of microseismic signals from coal seam roof hydraulic fracturing. ResNet, despite its skip connections effectively alleviating gradient vanishing and explosion in deep network training [27], is constrained by the local receptive fields of its convolutional operations, which restrict the modeling of long-range temporal dependencies within microseismic signals. VGGNet, with its architectural design featuring stacked small convolutional kernels and repeated max-pooling layers [28], suffers from a key drawback in that the downsampling process inherent to these operations results in the loss of fine-grained time-frequency details, such as subtle frequency shifts indicative of hydraulic fracturing dynamics, thereby compromising the ability of model to discern critical signal characteristics.

Zhang et al. [29] introduced a coal mine microseismic signal classification method based on the Swin Transformer network [30]. This approach directly converts temporal domain waveform signals into pixel histogram feature maps, which are then learned and trained by the Swin Transformer model, thus realizing the effective classification of microseismic and blast signals in coal mines. However, the aforementioned methods primarily focus on signals such as noise, blasting, and rock fracturing, and their specific effectiveness in identifying microseismic signals from hydraulic fracturing of coal seam roofs remains unknown. Notably, these methods lack explicit mechanisms to capture the fine-grained time-frequency distinctions between hydraulic fracturing and roof fracturing signals, which share highly similar morphological features but differ in subtle transient patterns.

In summary, existing microseismic identification methods reported in the literature primarily target signals with relatively low energy (e.g., weak microseismic events), high-energy signals (e.g., rock blast vibrations), and background noise. Most of these approaches rely on forward-modeled synthetic data rather than actual signals acquired from real mining environments, making them inadequate for automated identification of microseismic signals generated during hydraulic fracturing of coal seam roofs in practical coal mine applications. Based on the engineering background of roof hydraulic fracturing at the Yulin Caojiatan Coal Mine, this paper first analyzes the time-frequency characteristics of both hydraulic fracturing-induced microseismic signals and five categories of interfering signals, with a particular focus on discriminating between hydraulically fractured and roof-fractured microseismic signals that exhibit highly similar time-frequency features. After converting both types of signals into time-frequency feature maps via the FSWT transformation, these feature maps are input into a novel Swin Transformer-based identification model—chosen for its ability to model both local and global feature correlations, adapts to time-frequency data with different resolutions efficiently, and capture subtle transient patterns—to classify microseismic signals.

The major contributions of this paper are summarized as:

- Methodological Innovation: We propose a novel hybrid framework integrating FSWT and Swin Transformer for microseismic signal identification. FSWT enables precise time-frequency feature extraction via customizable frequency slice functions, while the Swin Transformer’s window-based attention mechanism effectively models both local details and global correlations in these features.
- Performance Advancement: The proposed method achieves an overall identification accuracy of 92.49% for the two target signal types on field-collected data from the Caojiatan Coal Mine, outperforming existing methods (energy distribution method: 64.70%, ResNet: 88.04%, VGGNet: 88.47%).
- Practical Significance: The research findings of this paper—based on real data collected from on-site coal mining operations—provide robust support for the localization of fracture networks and the evaluation of fracturing effectiveness subsequent to the hydraulic fracturing of coal seam roofs. Meanwhile, they also offer a scalable methodology for enhancing the monitoring of rockburst hazards in mining environments.

## 2. Engineering Background and Classification of Microseismic Events

### 2.1. Engineering Background

The Caojiatan Coal Mine is located in the Yushen Mining Area, northeast of Yulin City, with a mining area of approximately 108 km^2^ and proven resources of about 1.511 billion tonnes. Its approved production capacity is 25.0 Mt/a. The mine was designed with a production capacity of 17.0 Mt/a and currently extracts Coal Seam 2-2, which is minable across the entire area. The seam thickness ranges from 9.0 to 12.09 m, averaging 11.0 m, with burial depths between 255 and 338 m. The coal seam exhibits simple storage conditions. The immediate roof primarily consists of siltstone and fine sandstone, with a thickness ranging from 0.2 to 7.3 m. The main roof is composed of siltstone or medium sandstone, with individual layer thicknesses of 7–22 m. These strata exhibit underdeveloped bedding and are relatively hard. In-situ tests indicate that the siltstone has a compressive strength of 20–30 MPa, while the medium sandstone generally shows uniaxial compressive strengths exceeding 50 MPa. Additionally, the coal seam is overlain by multiple layers of thick and hard roof strata, each with a thickness of 10–35 m, thus defining the coal seam as an ultra-thick coal seam with exceptionally thick and rigid roof conditions.

**Figure 1:**
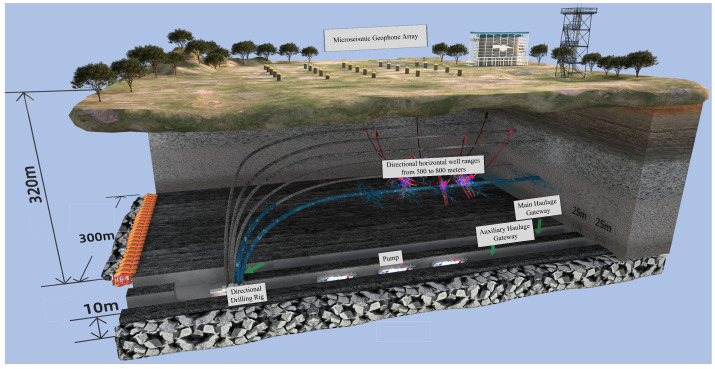
Schematic diagram of hydraulic fracturing and microseismic monitoring of coal mine roof.

Owing to the presence of thick and hard roof strata in the mine, strong rock pressure phenomena caused by the fracture of overlying rock layers during coal mining operations are particularly severe. Multiple incidents of large-scale strong roof weighting have occurred, resulting in widespread opening of support safety valves, instantaneous significant reduction in support height, and damage to support structural components under heavy dynamic loads. To ensure safe and efficient mining operations, regional hydraulic fracturing technology has been employed to weaken the extremely thick and hard rock strata, thereby reducing the intensity of rock pressure manifestations. This has led to the development of a targeted borehole regional fracturing technology system for strong rock pressure control at Caojiatan Coal Mine, which enhances the management technology level of thick and hard roof strata and provides guarantees for safe and efficient mining operations. During the fracturing process, the effectiveness of hydraulic fracturing is evaluated through comprehensive means including surface microseismic monitoring of fracture propagation range, quantitative analysis of high-energy roof fracture events, and evaluation of rock pressure manifestation intensity at the working face. The schematic diagram of the entire regional fracturing technology system is presented in Figure 1.

**Figure 2:**
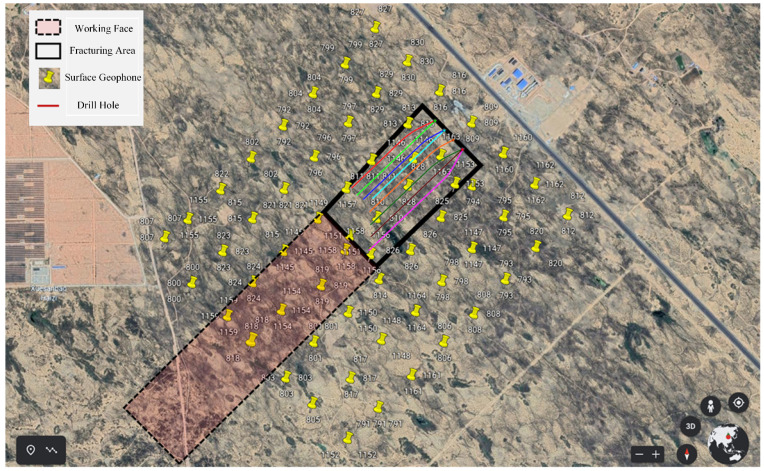
Satellite image of the arrangement of ground three component detectors.

The Caojiatan Coal Mine, with a burial depth of approximately 350 m, adopted a surface microseismic monitoring system composed of 60 three-component geophones. The deployment was optimized based on field surveys of topography, data reception quality, and noise sources. As shown in Figure 2, the geophones were installed at a depth of 0.2 m below the surface. The geophone spacing was designed to be 100–200 m, with localized adjustments made to ensure comprehensive coverage, optimal detection performance, and high positioning accuracy.

Microseismic waveform data were recorded in real time by the three-component geophones and the associated digital acquisition systems, then transmitted via 4G networks to cloud servers. The data could be downloaded to local computer hard drives in standard SEG-Y format with a time sampling interval of 60 s using acquisition control software. In field operations, the advance fracturing distance is generally controlled within a 500 m range. Therefore, when conducting surface microseismic monitoring in the fracturing area, geophones not only capture microseismic events induced by hydraulic fracturing but also capture microseismic signals from coal shearers and roof activities in the mining area. These interfering signals pose certain difficulties to the real-time microseismic monitoring of mine hydraulic fracturing, which differs significantly from the real-time microseismic monitoring in the petroleum industry. Consequently, the accurate identification and classification of various types of signals are the core issues to be resolved in the real-time microseismic monitoring technology for mine hydraulic fracturing.

### 2.2. Classification of Microseismic Events

During hydraulic fracturing of coal seam roofs, efficient and automatic recognition of various microseismic signals serves as the fundamental basis and essential prerequisite for accurate fracture network localization, roof stability evaluation, and disaster early warning. However, microseismic signals induced by numerous hydraulic fracturing events during monitoring are mixed with those from other microseismic sources (e.g., roof fracturing, coal cutting by shearers, underground directional drilling, distant mine tremors, and surface truck vibrations) [31]. These signals display similar time-domain characteristics and partially overlapping frequency-domain features.

Microseismic signals generated during hydraulic fracturing of coal seam roofs are characterized by relatively low amplitudes, with amplitudes typically less than 10 mV, and dominant frequencies ranging from 10 to 45 Hz. Distant mine tremors show clear separation of P- and S-waves due to long propagation distances, with dominant frequencies ranging from 10–15 Hz. Microseismic signals from shearer coal cutting are manifested as multiple repetitive signal groups within 60-s time intervals. Surface truck noise signals display a “spindle-shaped” waveform characterized by initial amplitude increase followed by gradual decay, accompanied by distinct Doppler effects in the frequency spectrum. Microseismic signals from underground drilling operations demonstrate persistent repetitive patterns. These four types of microseismic signals exhibit distinct waveform characteristics compared to hydraulic fracturing signals, enabling effective identification through conventional time-frequency analysis methods. However, roof fracture signals exhibit significant similarity to hydraulic fracturing signals in both waveform characteristics and frequency-domain overlap, with both typically having a duration of 2 to 3 cycles. This similarity renders the discrimination between these two types of signals particularly difficult. Microseismic signals induced by roof fracturing events pose the most severe interference to the accurate identification of hydraulic fracturing-induced microseismic signals. Therefore, this paper focuses on the automated recognition and classification of hydraulic fracturing microseismic signals and roof fracture microseismic signals.

## 3. Analysis of Microseismic Signal Characteristics

### 3.1. Frequency Slice Wavelet Transform (FSWT) Technique

FSWT introduces a scale factor δ and a frequency slice function $\hat{f}\left(\omega \right)$, enabling superior characterization of signals in the time-frequency domain. Compared to Fourier Transform, FSWT effectively captures localized frequency characteristics within specific time intervals—a capability unattainable with conventional Fourier-based methods. Furthermore, FSWT excels in feature extraction within designated intervals, making it particularly powerful for signal characterization tasks. In practical applications, FSWT can be implemented through a combination of Fast Fourier Transform (FFT) and Wavelet Transform, where FFT computes the signal’s frequency spectrum while Wavelet Transform analyzes its characteristics across different scales and frequencies. The computational complexity of this approach can be significantly optimized through FFT implementation, substantially enhancing computational efficiency.

#### 3.1.1. Forward FSWT

Let the wavelet basis function be f(t), and assume its FFT exists, denoted as the frequency slice function $\hat{f}\left(\omega \right)$. The mathematical expression for the FSWT of the signal $s\left(t\right)\in L^{2}\left(R\right)$ is then given by [32]:

(1) $$T_{f}\left(t,\omega ,\delta \right)=\frac{1}{2\pi }\gamma \int_{-\infty }^{+\infty }\hat{s}\left(u\right){\hat{f}}^{*}\left(\frac{\mu -\omega }{\delta }\right)e^{j\mu t}du$$

in which the symbol ∗ denotes the complex conjugate operation, $\hat{s}$ and $\hat{f}$ represent the FFT results of *s* and *f*, respectively. The scale factor δ is typically defined as a function related to *t*, ω, and μ, or alternatively specified as a nonzero constant.

Assuming γ=1, and based on the core concept of multiresolution analysis in Morlet wavelet transform, we introduce a time-frequency resolution coefficient κ, which is irrelevant to ω and μ, to adjust the time-frequency sensitivity of FSWT. Setting δ=ω/κ and substituting into the above Equation (1) yields:

(2) $$T_{f}\left(t,\omega ,\delta \right)=\frac{1}{2\pi }\int_{-\infty }^{+\infty }\hat{s}\left(u\right){\hat{f}}^{*}\left(\kappa \frac{\mu -\omega }{\omega }\right)e^{j\mu t}du$$

in which the time-frequency resolution coefficient κ can be obtained through the formula $\kappa =\Delta \omega_{f}/\eta_{s}$, where $\Delta \omega_{f}$ represents the frequency window width of the frequency slice function $\hat{f}\left(\omega \right)$ ($\hat{f}\left(\omega \right)=$ is selected as $\frac{1}{1+\omega^{2}}$ in this paper), and $\eta_{s}$ denotes the frequency resolution of the signal s(t). The time resolution decreases as the frequency resolution increases, and vice versa. Based on this principle, the objective of time-frequency multiresolution analysis is achieved. In the experiments conducted in this paper, to achieve a balanced time-frequency analysis performance, we employed the commonly used values for frequency window width $\Delta \omega_{f}$ ($\sqrt{2}/2$) and frequency resolution $\eta_{s}$ (0.025) [13,32]; consequently, the time-frequency resolution coefficient $\kappa =20\sqrt{2}$.

#### 3.1.2. Inverse FSWT

The inverse FSWT can be expressed in multiple forms, among which the most concise mathematical representation is given by:

(3) $$s\left(t\right)=\frac{1}{2\pi }\int_{-\infty }^{+\infty }\int_{-\infty }^{+\infty }T\left(\tau ,\omega ,\kappa \right)e^{i\omega (t-\tau )}d\tau d\omega$$

this equation indicates that the inverse FSWT is independent of the frequency slice function $\hat{f}\left(\omega \right)$. Signal reconstruction requires only an Inverse Fast Fourier Transform (IFFT) operation on the time-frequency surface obtained through FSWT. Furthermore, to extract the signal components of s(t) within specific time-frequency domains $\left(t_{1},t_{2},\omega_{1},\omega_{2}\right)$, it suffices to perform IFFT operations on the FSWT result T(τ,ω,κ) across the time domain $\left(t_{1},t_{2}\right)$ and frequency domain $\left(\omega_{1},\omega_{2}\right)$, expressed as:

(4) $$s_{t_{1},t_{2},\omega_{1},\omega_{2}}\left(t\right)=\frac{1}{2\pi }\int_{\omega_{1}}^{\omega_{2}}\int_{t_{1}}^{t_{2}}T\left(\tau ,\omega ,\kappa \right)e^{i\omega (t-\tau )}d\tau d\omega$$

as shown in Equation (4), after acquiring the time-frequency distribution of microseismic signals via FSWT, signal reconstruction can be implemented in accordance with different frequency bands. This allows for calculating the proportional distribution ratio of signals across frequency bands, thereby establishing a theoretical foundation for microseismic signal identification methods based on energy distribution characteristics [13].

### 3.2. FSWT-Based Energy Distribution of Microseismic Signals

The Frequency Slice Wavelet Transform (FSWT) technique enables the reconstruction of microseismic signals within arbitrary frequency ranges. Utilizing this feature, detailed sub-band analysis can be conducted on microseismic signals to calculate the energy distribution across sub-bands [13]. The energy calculation formula for signals in any frequency band is expressed as follows:

(5) $$E_{i,j}=\int_{T_{1}}^{T_{n}}s\left(t\right)dt=\sum_{k=1}^{n}{\left|s_{k}\right|}^{2}$$

where $E_{i,j}$ represents the energy value of the signal in the [i,j] Hz frequency band, $T_{1}$ and $T_{n}$ denote the starting and ending points in the time domain of the analyzed signal respectively, and $s_{k}\left(k=1,2,\ldots ,n\right)$ represents the amplitude values of sampling points within the [i,j]Hz frequency band during the $\left[T_{1},T_{n}\right]$ time interval. The total energy of the full-band signal is expressed as:

(6) $$E=\sum_{k=1}^{N}{\left|s_{k}\right|}^{2}$$

The percentage of sub-band energy relative to the total signal energy is calculated as:

(7) $$P_{i,j}=\frac{E_{i,j}}{E}\times 100\%$$

using this equation, the sub-band percentages for all hydraulic fracturing and roof fracture microseismic signals are computed separately. An optimal percentage threshold is then established to distinguish between these two types of microseismic signals. This FSWT-based energy distribution methodology for microseismic signals serves as one of the comparative methods, and its identification performance is detailed in Section 5.3.

### 3.3. Time-Frequency Domain Characteristics of Microseismic Signals

**Figure 3:**
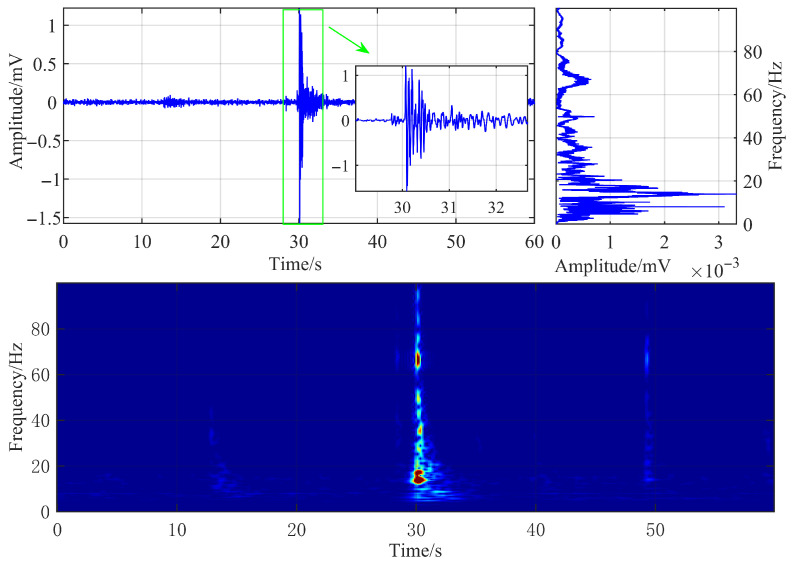
Waveform and time-frequency diagram of typical hydraulic fracturing microseismic signals.

A typical microseismic signal generated by hydraulic fracturing, captured by a surface three-component geophone at the Caojiatan Coal Mine, was selected as the research object for analysis. Its time-domain waveform, frequency spectrum, and time-frequency characteristics are illustrated in Figure 3.

The geophone sampling frequency was configured to 500 Hz. The hydraulic fracturing microseismic signal has a duration of approximately 4 s, characterized by an extended coda wave and slow attenuation of the waveform. The signal’s dominant frequency is approximately 14 Hz, and its energy is primarily concentrated in the frequency range below 20 Hz. By setting the time-frequency resolution coefficient $\kappa =\sqrt{2}/2/0.025=20\sqrt{2}$ and configuring the observation frequency range from 0 to 100 Hz, the time-frequency characteristic map was obtained via FSWT transformation.

**Figure 4:**
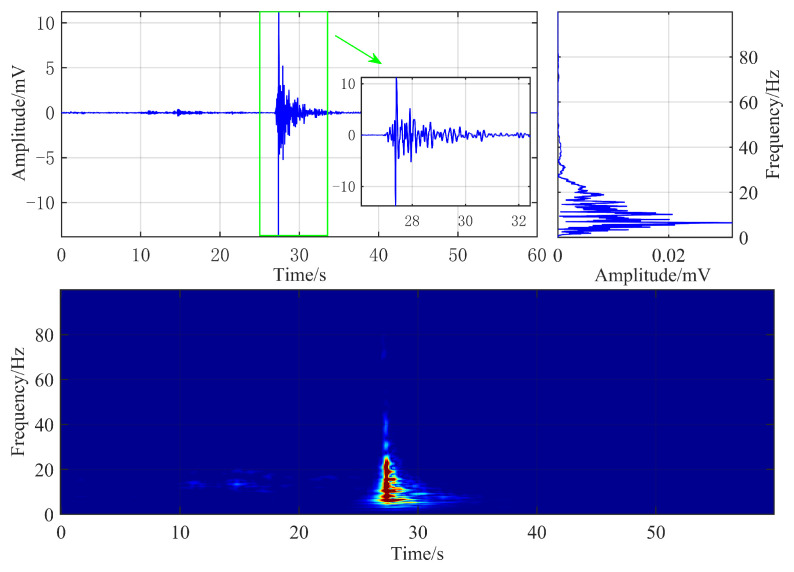
Waveform and time-frequency diagram of typical roof fracture microseismic signals.

The time-domain waveform, frequency spectrum, and time-frequency characteristics of a typical roof fracture microseismic signal are illustrated in Figure 4. The sampling frequency of the geophone was 500 Hz, and the roof fracture microseismic signal has a duration of approximately 5 s, characterized by a prolonged coda wave and slow waveform attenuation. The dominant frequency of the signal is approximately 6.5 Hz, and its energy is primarily concentrated in the frequency range below 18 Hz, exhibiting significant spectral overlap with hydraulic fracturing microseismic signals.

Hydraulic fracturing and roof fracturing microseismic signals exhibit obvious differences in their time-frequency representations. Based on this observation, this paper proposes to recast the microseismic signal recognition problem as a typical image recognition task. By utilizing the advanced Swin Transformer model to extract high-dimensional features from time-frequency images, accurate recognition of hydraulic fracturing microseismic signals can be realized.

## 4. Swin Transformer Based Automatic Identification Method for Microseismic Signals

### 4.1. Swin Transformer Based Automatic Identification Model

**Figure 5:**
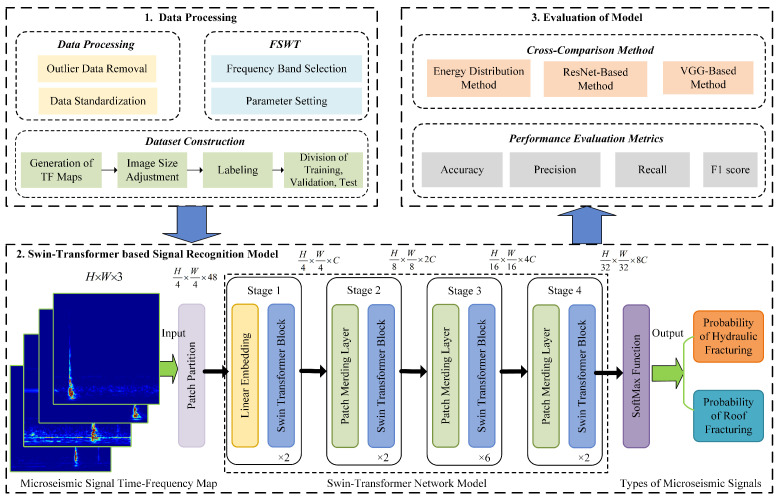
Schematic diagram of microseismic signal recognition based on Swin Transformer.

Figure 5 illustrates the principle of the microseismic signal recognition method based on Swin Transformer. The first step is data processing, which includes outlier data removal, data standardization, FSWT transformation, and dataset construction. The second step involves taking the time-frequency feature maps of microseismic signals obtained by FSWT transformation as training and validation datasets, inputting them into the Swin Transformer network model, and finally outputting the microseismic classification results through testing dataset. The third step is to evaluate the recognition performance of the model using metrics such as accuracy and F1-score, and compare its performance with other microseismic recognition methods.

Transformer, an attention-based architecture [33], excels in natural language processing (e.g., sequence modeling, translation) via its encoder’s self-attention, which models feature sequence relationships and enables cross-part information fusion. In computer vision, cross-region feature fusion boosts performance, leading to adapted Transformers like the representative Vision Transformer (ViT) [34,35].

The main structure of ViT is consistent with that of the standard Transformer encoder. First, the input time-frequency image of the microseismic signal is divided into multiple image patches. Each patch is then mapped into a one-dimensional vector using a linear embedding layer. To preserve the structural information of the time-frequency image before mapping, ViT also adds a position vector to each patch vector. Subsequently, the network structure follows the same encoder design as the traditional Transformer network. These image patches are input into multiple stacked Transformer blocks, and the refined features generated by the Multihead Self-Attention (MSA) module are used for subsequent tasks. The computation of MSA (taking single-head attention as an example) adopts the following formulas:

(8) $$Q={\mathrm{XW}}_{Q},\;K={\mathrm{XW}}_{K},\;V={\mathrm{XW}}_{V}$$

(9) $$Attention\left(Q,K,V\right)=Softmax\left(\frac{{\mathrm{QK}}^{T}}{\sqrt{d}}\right)V$$

where $X\in R^{p\times d}$ is the vector matrix of the input patches, *p* is the number of image patches, and *d* is the feature dimension. First, X is multiplied by three parameter matrices $W_{Q}$, $W_{K}$, and $W_{V}\in R^{d\times d}$ to obtain the query matrix Q, the key matrix K, and the value matrix *V*, respectively. Q and K are used to calculate the attention weight matrix ${\mathrm{QK}}^{T}$, which is then multiplied with the value matrix V to achieve enhanced image features.

Despite ViT’s strong performance in visual tasks, its large parameters and heavy pre-training data demands limit use in small-scale tasks. For microseismic time-frequency image classification, pre-training on natural image datasets (e.g., ImageNet) fails due to poor parameter transferability to microseismic data. Moreover, microseismic datasets are small —owing to scarce coarse-fine image pairs—further restricting large-parameter models.ViT’s large image patches (hundreds of pixels) also introduce redundant features, making it unsuitable for microseismic tasks that use tens-of-pixel images.

To address the aforementioned limitations of the ViT model, the Swin Transformer model was proposed in [30]. It employs a sliding window approach to improve computational efficiency, dividing the image into multiple non-overlapping windows, each containing small image patches of size *M*. This allows the self-attention module to be applied to the patches within each window. Under this window partitioning scheme, feature fusion occurs only in local regions of the image, and the pixel arrangement remains largely unchanged throughout the process, eliminating the need for a global position vector for each image patch as in ViT. However, considering that the self-attention module ignores the positional information of the computed image patches, it is still essential to introduce relative position information within the window. Therefore, Swin Transformer incorporates a relative positional bias in the self-attention module, as shown in the following formula:

(10) $$Attention\left(Q,K,V\right)=Softmax\left(\frac{{\mathrm{QK}}^{T}}{\sqrt{d}}+B\right)V$$

where $B\in R^{M^{2}\times M^{2}}$ is the relative position bias, representing the relative position information between image patches within a single window. Swin Transformer’s Window-based Multihead Self-Attention (W-MSA) reduces computational complexity vs. ViT’s MSA but lacks cross-window information, limiting feature expression. It thus adds Shifted Window-based Multihead Self-Attention (SW-MSA) for cross-patch feature fusion via consecutive attention blocks. To address the linear receptive field growth of these blocks, a patch merging layer reduces resolution, expands the field of view, and enables multi-scale (coarse-fine) feature extraction from microseismic time-frequency images. This paper uses Swin Transformer for accurate identification of such images.

**Figure 6:**
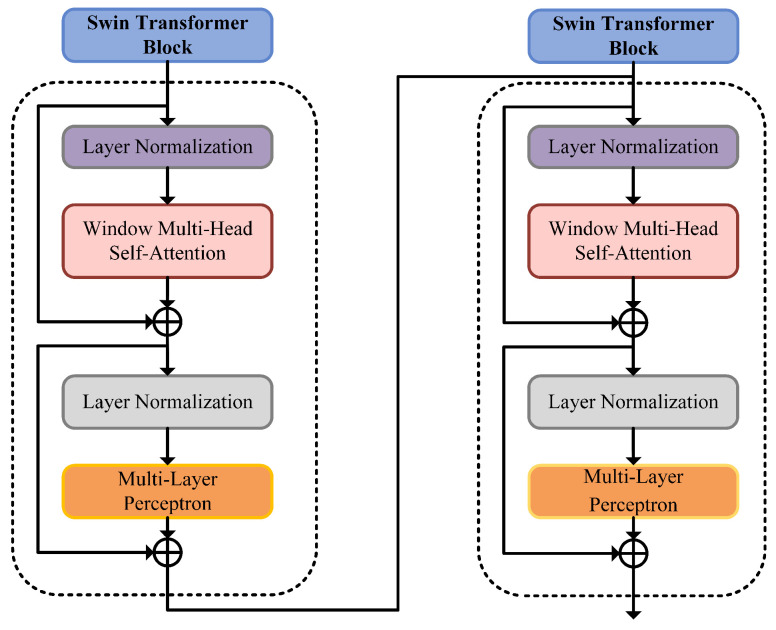
Structure diagram of Swin Transformer block.

The overall structure of the microseismic signal identification model (as shown in the lower part of Figure 5) is consistent with that of the Swin Transformer [35]. First, the time-frequency characteristic image set of microseismic signals obtained through data preprocessing and FSWT transformation is input into the patch segmentation layer for partitioning. The RGB image is partitioned into non-overlapping image patches. Each adjacent 4×4 pixels form a patch, which is then flattened along the channel dimension. Since the input is an RGB three-channel image, each patch has 16 pixels, and each pixel has R, G, B three values, so the flattened size is 48. More generally, after passing through the patch segmentation layer, the image size changes from H×W×3 to $\frac{H}{4}\times \frac{W}{4}\times 48$. Then, a linear embedding layer performs a linear transformation on the image along the channel dimension, changing the size from 48 to *C*, i.e., the image size changes from $\frac{H}{4}\times \frac{W}{4}\times 48$ to $\frac{H}{4}\times \frac{W}{4}\times C$. Subsequently, feature maps with different resolutions are generated through four stages. With the exception of the first stage, which first undergoes a linear embedding layer, the remaining three stages first perform downsampling via a patch merging layer, followed by passing through two Swin Transformer blocks stacked repeatedly, as shown in Figure 6.

Finally, after passing through the SoftMax activation function, the probabilities of hydraulic fracturing and roof fracture microseismic signals are predicted, with the type having the higher probability value being the final predicted microseismic signal type.

### 4.2. Microseismic Dataset

The dataset used in this paper consists of real microseismic signals collected on-site from the Caojiatan Coal Mine. A large number of coal seam roof hydraulic fracturing microseismic events and roof fracturing microseismic events were recorded using an array of 60 high-precision three-component geophones deployed on the ground at the Caojiatan Coal Mine in Yulin. It should be noted that the microseismic dataset was collected by professionals during and immediately after hydraulic fracturing in coal mines, and manually labeled with microseismic categories.

#### 4.2.1. Data Preprocessing

The sampling rate of the geophone is 500 Hz, which is sufficient to capture all frequency components of microseismic signals with a dominant frequency of only several tens of Hz. Each microseismic signal was truncated to a fixed length of 60 s (corresponding to 30,000 data points) based on the statistical duration of typical hydraulic fracturing and roof fracture events in the field, ensuring uniform input dimensions for the model.

Denoising: Field-collected microseismic signals are inevitably contaminated by environmental noise (e.g., surface traffic, mechanical vibration) and electromagnetic interference. To improve the signal quality, a clipping process was first performed: based on the frequency range analysis of the target microseismic signals (<100 Hz), the frequency components above 100 Hz were clipped off.

DC Removing: A direct current (DC) component removal operation was applied to the denoised microseismic signals. This step aimed to eliminate the baseline drift caused by long-term sensor offset or environmental temperature fluctuations, which could otherwise distort the amplitude characteristics of the target signals. The DC component was estimated as the mean value of the truncated signal over its entire time series and subtracted from each data point, ensuring that the signal oscillated around a zero baseline.

#### 4.2.2. Dataset Division

In total, the dataset contains 9090 microseismic signals, including 4545 hydraulic fracturing signals and 4545 roof fracture signals. These signals were divided into training, validation, and test sets in a ratio of about 6:2:2 (5400, 1800, and 1890 samples, respectively). The training set was used to optimize network parameters, the validation set to guide hyperparameter adjustment and prevent overfitting or underfitting, and the test set to evaluate the model’s predictive capability.

To rigorously verify the generalization ability of the proposed Swin Transformer recognition model on newly acquired microseismic signals, the traditional random allocation method was not adopted. Instead, signals were sorted by acquisition timestamps and split in about 6:2:2 ratio. Specifically, signals collected in the earlier period were used for training, and those collected in the later period for validation and testing. This method of sorting by time and then splitting directly simulates real-world application scenarios to verify that the model’s generalization ability can reflect its actual performance in engineering practice.

### 4.3. Training and Testing of the Microseismic Signal Identification Model

#### 4.3.1. Training Phase of the Model

The training dataset and its corresponding labels are employed to optimize the parameters of the Swin Transformer model. These parameters include the weights w and biases b of individual neurons, as well as the query matrix Q, key matrix K, and value matrix V in the self-attention mechanism. The training process of the identification model is essentially an iterative optimization process where neural network parameters are automatically updated based on gradient information to minimize the loss function. For binary classification problems, the most commonly adopted loss function is the Cross-Entropy function. This function quantifies the discrepancy between predicted results and ground truth labels by computing the product of the logarithmic probability of true labels and the predicted probability. The mathematical expression for binary cross-entropy loss is expressed as:

(11) $$CrossEntropy=-\frac{1}{N}\sum \left(y_{i}\log\left({\hat{y}}_{i}\right)+\left(1-y_{i}\right)\log\left(1-{\hat{y}}_{i}\right)\right),$$

where *N* denotes the number of training samples, $y_{i}$ represents the ground-truth label of the *i*-th sample (e.g., the hydraulic fracture microseismic signal is labeled as 1, while the roof fracture microseismic signal is labeled as 0), and ${\hat{y}}_{i}$ denotes the predicted probability of the *i*-th sample. After setting the initial hyperparameters of the network model (such as learning rate γ, batch_size, number of epochs, activation function, and the number of network layers), the microseismic signals are sequentially fed into the Swin-Transformer model in the form of time–frequency images. Subsequently, the network parameters are updated using the stochastic gradient descent (SGD) algorithm to minimize the loss function.

**Figure 7:**
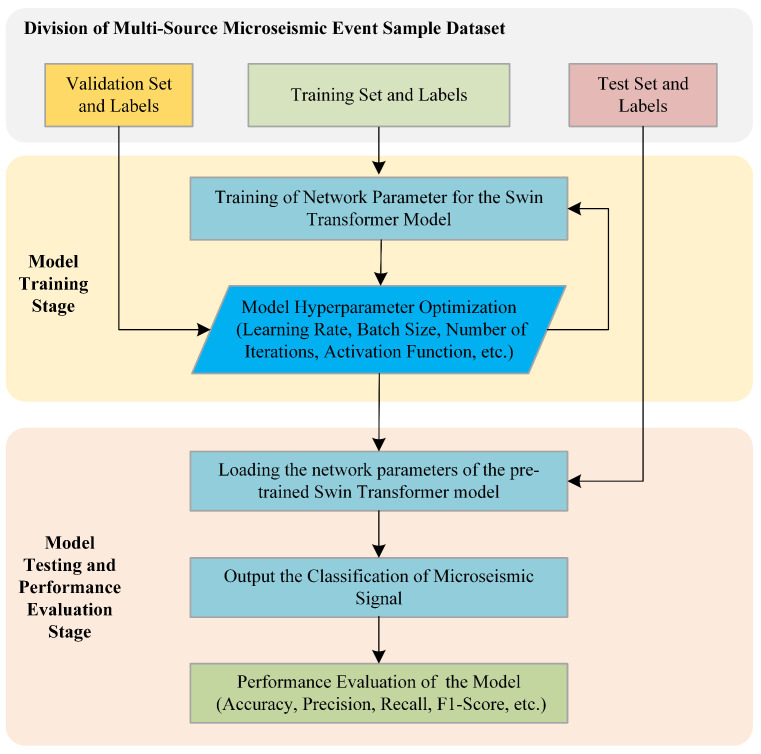
Training and testing flow chart of microseismic signal recognition model.

The function of the validation dataset and its labels is to guide the optimization of the network model’s hyperparameters, with an input process consistent with that of the training set. If, during training, the loss function value continuously decreases on the training set but shows a decreasing-then-increasing trend on the validation set, this indicates overfitting, i.e., the model performs well on the training set but fails to generalize on the validation set due to excessive focus on detailed features. Conversely, if the loss function value decreases steadily on the training set but fails to converge on the validation set, this suggests underfitting. This means that the model fails to learn sufficiently deep-level features during training and that network hyperparameters need adjustment. After the model achieves the expected performance on both the training and validation datasets, the parameters and hyperparameters of the Swin Transformer model are finalized, see Figure 7.

#### 4.3.2. Testing Phase of the Model

In the testing phase of the model, the fixed network parameters and hyperparameters of the Swin Transformer are first loaded. Subsequently, the time–frequency spectrograms of all microseismic signals in the test set are input into the model in a single batch to generate the predicted category for each signal. Finally, the predicted categories are compared with the ground-truth labels, and a statistical analysis is conducted. The recognition performance of the model is comprehensively evaluated using four metrics: accuracy, precision, recall, and F1-score, the definitions of which are described in detail in the subsequent section.

## 5. Results and Analysis

### 5.1. Evaluation Metrics

In machine learning and statistics, the confusion matrix is widely employed to evaluate the performance of classification models. It provides an intuitive visualization of the classification results in a matrix form by comparing the model’s predictions with the actual labels, making it particularly suitable for classification tasks in supervised learning. The basic structure of a confusion matrix is presented in the following Table 1:

In the confusion matrix, True Positive (TP) refers to the number of positive samples that are correctly predicted as positive samples. False Negative (FN) refers the number of positive samples that are erroneously predicted as negative samples. False Positive (FP) refers the number of negative samples that are erroneously predicted as positive samples. True Negative (TN) refers to the number of negative samples that are correctly predicted as negative samples.

By classifying the model’s classification results into true cases (True) and false cases (False), a set of performance metrics can be quantitatively calculated, including accuracy, precision, recall, and the F1-score. These metrics comprehensively assess the classification capability of the model.

Accuracy is a comprehensive overall performance metric that quantifies the ratio of correctly predicted samples to the total number of samples. It is formally defined as:

(12) $$Accuracy=\frac{TP+TN}{TP+TN+FP+FN}.$$

Precision quantifies the proportion of correctly predicted positive samples among all samples predicted as positive. It is defined as:

(13) $$Precision=\frac{TP}{TP+FP}.$$

Recall, also referred to as sensitivity, measures the proportion of correctly predicted positive samples among all actual positive samples. It is calculated as:

(14) $$Recall=\frac{TP}{TP+FN}.$$

The F1-score is the harmonic mean of precision and recall, providing a balanced measure of both metrics. It is expressed as:

(15) $$F1=2\times \frac{Precision\times Recall}{Precision+Recall}.$$

The F1-score, which ranges from 0 to 1, serves as a unified metric for evaluating model performance, where values closer to 1 indicate better performance. It is defined as the harmonic mean of precision and recall, thereby explicitly balancing the trade-off between these two metrics. The F1-score is particularly informative for binary classification problems, especially when an equilibrium between precision and recall is desired. A high F1-score indicates that the model achieves an optimal balance, maintaining both high precision and high recall.

### 5.2. Results of the Proposed Method

During the training phase of the Swin Transformer model, the hyperparameters were iteratively tuned based on the model’s performance on both the training and validation datasets. After extensive experimental validation, the optimal hyperparameter configuration was finalized, with the batch_size configured to 16 and the learning rate γ configured to 0.0002, thereby enabling the model to achieve excellent recognition performance.

**Figure 8:**
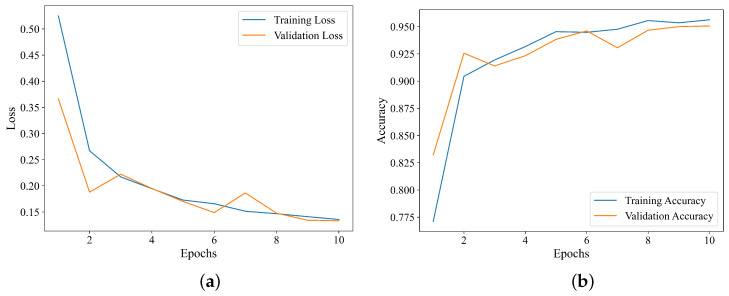
Curves of loss and accuracy vs. epochs during model training. (**a**) Relationship between loss and epochs. (**b**) Relationship between accuracy and epochs.

As illustrated in Figure 8a, during the training of the Swin Transformer model, the loss function values on both the training and validation datasets show an overall downward trend with the increase in the number of epochs. This phenomenon indicates that the Swin Transformer network is neither underfitted nor overfitted. Figure 8b demonstrates that, as the number of epochs increases, the overall recognition accuracy of the model for hydraulic fracturing and roof fracture microseismic events rises on both the training and validation datasets, with the curve eventually stabilizing at a plateau. Considering both recognition performance and computational efficiency comprehensively, the value of epochs was configured to 5 in this paper.

**Figure 9:**
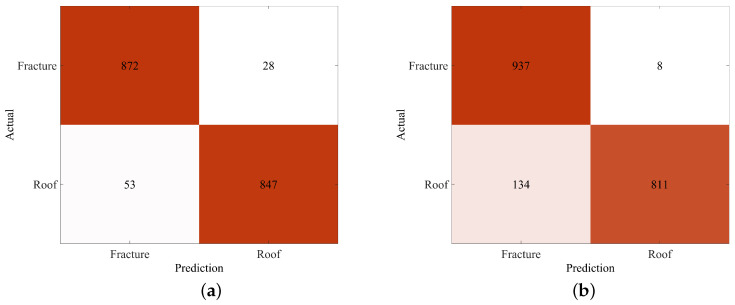
The confusion matrix of the model on the validation and test sets. (**a**) Confusion matrix on the validation set. (**b**) Confusion matrix on the test set.

Figure 9 presents the microseismic event recognition results of the Swin Transformer model on both the validation and test datasets. The confusion matrix reveals that, in the validation set, among 900 actual hydraulic fracturing (sbbreviated as fracture) microseismic signals, 872 were correctly predicted as fracture signals, while 28 were misclassified as roof fracture (abbreviated as roof) signals. For the 900 actual roof microseismic signals, 847 were correctly identified as roof signals, whereas 53 were incorrectly classified as fracture signals. In the test set, among 945 actual fracture microseismic signals, 937 were correctly predicted as fracture signals, with 8 misclassified as roof signals. For the 945 actual roof microseismic signals, 811 were correctly identified as roof signals, while 134 were misclassified as fracture signals. According to the evaluation metrics defined in Section 5.1, the recognition accuracy, precision, recall, and F1-score for hydraulic fracturing microseismic signals are 92.49%, 87.49%, 99.15%, and 92.96%, respectively, which verifies the effectiveness of the proposed model.

To further validate the connection between the predictions of model and input features, we applied Gradient-weighted Class Activation Mapping (Grad-CAM) to the Swin Transformer. Through the model’s hierarchical feature maps, gradients of the classification scores were backpropagated to the input FSWT spectrogram features, generating heatmaps where high-intensity regions (red-yellow) indicate areas most critical to the model’s decision-making.

**Figure 10:**
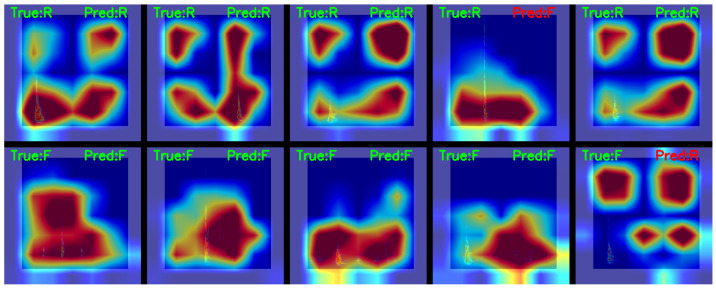
Grad-CAM heatmaps overlaid on the original FSWT spectrograms.

Figure 10 presents Gradient-weighted Class Activation Mapping (Grad-CAM) heatmaps overlaid on the original FSWT spectrograms, featuring 5 roof fracturing microseismic signals (the 5 subfigures in the upper panel) and 5 hydraulic fracturing microseismic signals (the 5 subfigures in the lower panel), with F denoting fracturing and R denoting roof fracture. The heatmaps explicitly demonstrate that the model discriminates between the two signal types by focusing on time-frequency continuity (for hydraulic fracturing) and time-frequency discontinuity (for roof fracturing), with particularly distinct differences. This aligns with the intrinsic characteristics of these microseismic events: Hydraulic fracturing operations are continuous processes, where the coal seam roof is gradually fractured as water pressure increases, exhibiting temporal continuity in signal features.

Additionally, Figure 10 also presents the Grad-CAM heatmaps of misclassified hydraulic fracture and roof fracture spectrograms. For roof fracture signals with relatively low energy (low signal-to-noise ratio), their Grad-CAM maps exhibit time-frequency continuity, and the model focuses on the medium-low frequency regions throughout the entire signal duration, thus being misidentified as “hydraulic fracturing” by the model. Conversely, for hydraulic fracturing signals with relatively high energy, their Grad-CAM maps show time-frequency discontinuity. The model pays attention to the low-frequency and high-frequency regions during the event and in the time periods before or after the event, highlighting the suddenness of the event, and thus is misidentified as “roof fracturing” by the model.

The qualitative visualization—Grad-CAM heatmaps—demonstrates that the decision-making process of Swin Transformer is grounded in physically meaningful features of the microseismic signal. Its hierarchical attention mechanism, enabled by shifted windows, allows for progressive refinement of focus from low-level to high-level discriminative features, while class-specific attention biases align with domain knowledge of hydraulic fracturing and roof fracturing events. These results not only enhance the interpretability of the model but also reinforce the credibility of its quantitative performance metrics, confirming that the proposed method is both effective and mechanistically sound.

### 5.3. Comparative Analysis

In this paper, several microseismic signal recognition methods reported in existing literature are implemented, including the signal energy distribution method [13], the ResNet-based recognition method [21], and the VGGNet-based recognition method [22]. The principle of the signal energy distribution method is described in detail in Section 3.2. The core of the ResNet model resides in the residual block, which consists of two convolutional layers. The output of the second convolutional layer is combined with the input to form a crucial connection that enables the block to collect and learn residual information. Finally, classification is performed through fully connected layers. In this paper, the ResNet18 model, identical to that employed in [21], is utilized. This model contains a total of 18 convolutional and fully connected layers, and its substantial depth allows the network to capture and learn increasingly complex and abstract features. Regarding the VGGNet-based recognition method, this paper employs the deeper VGG11 model instead of the VGG4 adopted in [22]. The VGG11 model is composed of 11 hidden layers, including 8 convolutional and pooling layers, as well as 3 fully connected layers. Using the same microseismic signal dataset, experimental analyses are conducted on the aforementioned three comparative methods, and their performance is compared with that of the proposed Swin Transformer-based recognition method, as shown in Table 2.

As shown in Table 2, the recognition accuracy of the signal energy distribution method for hydraulic fracturing and roof fracture microseismic signals were only 64.70% and 63.35%, respectively, indicating its limited robustness in practical applications. The ResNet18 and VGGNet models achieved recognition accuracies of 80.48% and 83.47%, respectively, with corresponding F1-scores of 80.41% and 83.50%. In contrast, the proposed Swin Transformer model significantly outperformed the baseline methods, achieving recognition accuracies of 92.49% and 92.96% for hydraulic fracturing and roof fracture microseismic signals, respectively, and an F1-score of 92.96% for hydraulic fracturing microseismic signals. These results clearly verify the superior performance of the proposed Swin Transformer-based recognition method.

## 6. Conclusions

This paper focused on addressing the challenge of extracting hydraulic fracturing microseismic signals under multi-source interference during the hydraulic fracturing of extremely thick coal seams at Caojiatan Mine, Yulin. Through time-frequency domain analysis of five types of interference signals (remote mine tremors, shearer coal cutting, surface truck noise, directional drilling, and roof fracture) and target hydraulic fracturing signals, we identified a critical insight: while most interference signals differed significantly from hydraulic fracturing signals in time-frequency characteristics, roof fracture signals shared striking similarities—including P- and S-wave separation, almost identical durations, substantial spectral overlap, and partially overlapping energy ranges. This finding confirmed that the primary technical bottleneck lies in the automatic and accurate discrimination between hydraulic fracturing and roof fracture microseismic events.

To resolve this bottleneck, we proposed a hybrid recognition model integrating FSWT and Swin Transformer. FSWT was employed to transform hydraulic fracturing and roof fracture signals, with a custom frequency-slicing function enabling synchronous time-frequency feature extraction and generating spectrograms that preserved fine-grained local signal characteristics. The Swin Transformer network was then trained and optimized using a dataset of these field-collected microseismic data. Validation results showed the model achieved an overall classification accuracy of 92.49% on the test set, significantly outperforming the energy spectrum division method, ResNet-based method, and VGGNet-based method. Beyond superior recognition performance, the model also demonstrated strong generalization to new field-collected data, providing a reliable technical solution for the automatic identification of coal seam roof hydraulic fracturing microseismic signals in practical mining operations.

## Figures and Tables

**Table 1:** Structure of Confusion Matrix.

	Predicted Positive	Predicted Negative
Actual Positive	True Positive (TP)	False Negative (FN)
Actual Negative	False Positive (FP)	True Negative (TN)

**Table 2:** Comparison of hydraulic fracturing microseismic signal recognition performance with other methods.

Method	Accuracy	Precision	Recall	F1-Score
Energy spectrum division method	0.6470	0.6355	0.6470	0.6412
ResNet-based method	0.8048	0.8134	0.8048	0.8041
VGGNet-based method	0.8347	0.8402	0.8347	0.8350
Swin Transformer-based method (proposed)	0.9249	0.8749	0.9915	0.9296

## Data Availability

The datasets presented in this article are not readily available because the data are part of an ongoing project. Requests to access the datasets should be directed to wpeng1205@sina.com.
